# Cytotoxic activity evaluation of chalcones on human and mouse cell lines

**DOI:** 10.1186/1753-6561-8-S4-P52

**Published:** 2014-10-01

**Authors:** Luis Felipe Buso Bortolotto, Bruna Cestari Azevedo, Gabriel Silva, Mozart Marins, Ana Lucia Fachin

**Affiliations:** 1 Universidade de Ribeirão Preto, Brazil

## Background

The Brazilian National Cancer Institute (INCA) estimated last year almost forty thousand new cancer cases of laryngeal carcinoma, melanoma, tracheal, bronchial and lung cancer. It is known that multidrug resistance and unspecific toxicity are the major challenges for commercial anticancer drugs [1]. In this regard, natural compounds and derivatives are considered as a source of novel antitumor drugs. Among these molecules, chalcones have gained pharmacological importance due its mechanisms of action related to apoptosis induction [2]. Here we characterized the cytotoxicity of four chalcones toward three tumor cell lines: Hep-2 (human laryngeal squamous carcinoma cells), B-16 (mouse melanoma), A549 (human lung adenocarcinoma epithelial cells) and one normal cell line: 3T3 (mouse fibroblasts).

## Methods

The cytotoxicity of chalcones was evaluated by the colorimetric MTT assay, which determines the reduction of tetrazolium into insoluble formazan by mitochondria of viable cells [3]. For this purpose, four chalcones: *trans*-chalcone; Licochalcone A; 4-Methoxychalcone and 3'-(trifluoromethyl)chalcone were tested against four different cell lines: Hep-2, B-16, A549 and 3T3. The cell culture was carried out by using Dulbecco's Modified Eagle Medium for A549 and 3T3, RPMI 1640 for Hep-2, and F-10 for B-16. All medium were supplemented with fetal calf serum. Each cell line was plated in a 96-well plate (3x10^4 ^per well) for 24 hours before adding the medium with chalcones in a 5 different concentration treatment (25, 20, 15, 10 and 5 µg/mL). After 48 hours, compounds were withdrawn and a solution of fresh medium containing 3-(4,5-dimethylthiazol-2-yl)-2,5-diphenyltetrazolium bromide (MTT) was added. After 4 hours of incubation, the product (formazan) was solved by DMSO. Hereafter, the plates were measured through 550nm wavelength analysis, using an ELISA reader. Treatments were compared to negative control (medium with 0.25% DMSO) and positive control (2.5 µg/mL doxorubicin). Cytotoxicity was calculated by the formula: percent cytotoxicity = (1-[absorbance of experimental wells/absorbance of control wells])×100% [4]. IC_50 _values were also determined. Data were analyzed by the software *Sisvar*.

## Results and conclusions

The IC_50 _values for A549 were 81.29 µM, 81.34 µM, 85.40 µM and 46.13 µM for *trans*-chalcone, 3'-(trifluoromethyl)chalcone, 4-Methoxychalcone and Licochalcone A, respectively. Hep-2 showed up the most susceptible cell line with IC_50 _values below 10 µg/mL for *trans*-chalcone, Licochalcone A and 3'-(trifluoromethyl)chalcone. IC_50 _values for Licochalcone A, *trans*-chalcone, 4-Methoxychalcone and 3'-(trifluoromethyl)chalcone for tumor mouse cell line B-16 were, respectively, 25.89 µM, 45.42 µM, 50.15 µM and 61.54 µM; for non-tumor mouse cell line 3T3 were 33.42 µM, 48.40 µM, 64.34 µM and 43.44 µM. All IC_50 _values for 4-Methoxychalcone were higher than 50 µM. Thus, among the chalcones tested 4-Methoxychalcone was the least cytotoxic and Licochalcone A the most effective for all cell lines, followed by *trans*-chalcone and 3'-(trifluoromethyl)chalcone.
